# “Bupe by the Book”: A study protocol for a pilot randomized controlled trial of library-facilitated telehealth to increase buprenorphine treatment among unstably housed individuals

**DOI:** 10.21203/rs.3.rs-5507141/v1

**Published:** 2024-11-25

**Authors:** Lianne A. Urada, Carla Marienfeld, Megan Partch, Richard S. Garfein, Steffanie A. Strathdee, Melanie J. Nicholls, Ashley Weitensteiner, Maria Luisa Zuniga, Peter Davidson, Eileen Pitpitan

**Affiliations:** University of California San Diego School of Medicine and San Diego State University School of Social Work; University of California San Diego School of Medicine; Father Joe’s Villages, Village Health Center; UC San Diego, Herbert Wertheim School of Public Health and Human Longevity Science; University of California San Diego School of Medicine; San Diego State University School of Social Work; University of California San Diego School of Medicine and San Diego State University School of Social Work; San Diego State University School of Social Work; University of California San Diego School of Medicine; University of California San Diego School of Medicine and San Diego State University School of Social Work

**Keywords:** Homelessness, unhoused, unstably housed, opioid use, opioid use disorder, buprenorphine treatment, substance use treatment, public libraries, randomized controlled trial

## Abstract

**Background:**

Accessing opioid use disorder (OUD) treatment is difficult for individuals in unstable housing. This population often uses public libraries for computer and internet access, which could provide telehealth access to OUD treatment. Therefore, we developed a novel 12-week library-facilitated telehealth intervention study called “Bupe by the Book” (BBB), which uses library resources to facilitate the initiation and retention of OUD treatment with buprenorphine.

**Methods:**

This study involved a partnership between the San Diego Public Library and a federally qualified healthcare center attached to a homeless shelter (Father Joe’s Villages (FJV) Village Health Center). We codesigned a pilot randomized controlled trial to evaluate a library-facilitated telehealth intervention in San Diego, California. We evaluated the intervention for its feasibility and acceptability and to obtain an estimate of the effect of the intervention on buprenorphine treatment outcomes. Individuals reporting homelessness and OUD (with or without other substance use) were eligible. Forty library patrons were recruited via flyers, screened for eligibility, and referred to the FJV Health Center for initial buprenorphine treatment intake visits. Participants who completed intake were enrolled and randomized to the library-facilitated telehealth condition, which involved the use of library internet and computer resources for follow-up visits to the clinic for buprenorphine treatment. The participants completed treatment follow-up in person or by phone in the control condition. Planned analyses (not powered to assess efficacy) will provide effect size estimates of the library-facilitated telehealth intervention on 1) buprenorphine use (measured in weekly urine drug screenings), 2) use of illicitly manufactured fentanyl (IMF) and other opioids (measured in weekly urine drug screens), 3) number of buprenorphine prescription pick-ups, 4) number and frequency of clinic visits, and 5) self-reported substance use, mental health, and quality of life measures at 1, 2, 4, 8, and 12 weeks.

**Discussion:**

The findings from this pilot study may support the adoption of library-facilitated telehealth treatment as a feasible and acceptable strategy to engage and retain unstably housed people with OUD in buprenorphine treatment. The lessons from this pilot study include the importance of community–academic partnerships in sustainably adapting interventions in community-based settings.

**Trial registration:**

This trial was registered prospectively at ClinicalTrials.gov (registration number NCT05872386) on May 24, 2023.

## Background

Recently, the number of overdose deaths in the U.S. has increased by as much as 30% compared with that reported in prior years [1]. In San Diego County, California, there were 814 fentanyl-related deaths in 2022 compared with 462 in 2020 [2]. In the fight against opioid use disorder (OUD), buprenorphine has been used nationally as the first promising medication that can be prescribed or dispensed by physicians in medical offices [3].

However, formidable barriers to buprenorphine uptake remain challenging, including uptake among persons who are unstably housed [4]. In 2023, overdose deaths decreased nationally by three percent for the first time since 2018 [5]. However, the number of overdose deaths among unhoused individuals in San Diego increased 200% from 2019–2023 [6]. Unhoused people are disproportionately impacted by the overdose crisis, accounting for 38.7% of reported overdose deaths across San Diego County in 2022 [6, 7].

In the U.S., over half a million people experience homelessness [8]. The city of San Diego, California, has the fifth largest homeless population nationally [9] and a rapidly increasing population with OUD [10]. Nearly 30% of unstably housed persons struggle with mental health and substance use disorders [11]. Many people with OUD remain unhoused and profoundly hard to reach.

However, one place to find unstably housed persons is the public library. Nationwide, public libraries face an on-premise opioid overdose crisis, especially among their unstably housed patrons. Data from our formative research in San Diego revealed that an estimated “two overdoses a day” occur just outside the library among those experiencing homelessness and other harms [12–17]. Libraries offer a safe place for many unhoused individuals during the day, and numerous libraries have already taken extraordinary measures to help unstably housed patrons with OUD [18, 19]. Some libraries now have onsite social workers, nurses, and peer homeless outreach staff [20–32]. In the downtown San Diego Central Library, library security staff were trained to carry and administer naloxone to patrons who overdosed in the bathrooms and around the immediate vicinity. Because unstably housed individuals with OUD regularly patronize public libraries, a public health opportunity presents itself to offer OUD treatment.

Buprenorphine is a highly effective chemotherapeutic treatment for OUD that has been shown to reduce the risk of opioid overdose [33]. Buprenorphine uptake and adherence may depend on an individual’s housing status and other issues impacting their ability to participate in treatment. Typically, patients follow an individualized plan that includes visits for medication management, counseling sessions, and urine drug screenings. However, our preliminary work showed that unstably housed persons with OUD at libraries report barriers to following this plan, including a lack of transportation for in-person visits, a lack of smartphones or computers for telehealth encounters, and a lack of consistent telephone numbers/devices for telephone visits [13–14]. Other barriers to uptake and adherence, such as stigma, lack of knowledge of medications, high out-of-pocket costs, inconsistent insurance coverage, and ethnic disparities related to medication access, are well documented [34–42].

Telehealth interventions for buprenorphine, delivered per national standards, provide patients with buprenorphine prescription access through remote telehealth (audio and video or even audio only) sessions with a prescribing medical provider without having to visit in-person clinics as frequently or ever. Research shows that telehealth interventions for buprenorphine effectively overcome barriers to buprenorphine uptake/adherence [43]. After the U.S. Drug Enforcement Administration relaxed the requirements for prescribing buprenorphine in March 2020 (during the COVID-19 pandemic), studies began highlighting low-barrier buprenorphine treatment options as more accessible under national regulatory changes [44, 45]. The studies included unhoused persons in San Francisco [46], mobile buprenorphine/case management for homeless veterans [47], and a van-based buprenorphine induction clinic in partnership with a federally qualified health center [48]. A review of telehealth-delivered medications for OUD revealed that a videoconference intervention led to better substance use treatment retention than did an in-person intervention [49–52]. These studies suggest that telehealth can promote the initiation of buprenorphine.

However, we must learn more about how telehealth can improve OUD treatment outcomes for unstably housed populations. A recent study revealed disadvantages for those with digital literacy barriers [53]. Unstably housed persons are less likely to have reliable interconnectivity [54]. As such, libraries present themselves as public health opportunities to facilitate telemedicine [55] by providing computers, high-quality internet connections, and private spaces where patrons can conduct confidential telehealth calls.

This protocol describes our “Bupe by the Book” (BBB) study, which is being conducted in collaboration with established local partners, including San Diego Public Libraries and Father Joe’s Villages - Village Health Center (FJV), a federally qualified healthcare center (FQHC). Previously, FJV provided patients with an option for tele-buprenorphine (telehealth for buprenorphine) for those who reported access to a device (e.g., computers, smartphones, tablets, or telephones). Representing a significant step further, our study (BBB) eliminates the need for access to a device. Instead, it leverages public library resources (devices and the internet) to allow unstably housed individuals to access telehealth care for buprenorphine. BBB is a groundbreaking intervention evaluated via a 12-week, two-arm randomized controlled trial (RCT) design. By testing library-facilitated telehealth, this study reveals how unstably housed persons and others can receive buprenorphine care innovatively with minimal clinical/nonclinical requirements (e.g., low-barrier induction offered without a waiting period).

## Methods

### Objectives and hypotheses

The overall objective of this study was to determine the feasibility and acceptability of utilizing library-facilitated telehealth to provide buprenorphine access to unstably housed people with OUD. As a pilot study that is not statistically powered to assess efficacy, the study will provide effect size estimates of the library-facilitated telehealth BBB intervention on 1) buprenorphine use (measured in weekly urine drug screenings), 2) use of illicitly manufactured fentanyl (IMF) and other opioids (measured in weekly urine drug screens), 3) buprenorphine prescription pick-ups, 4) number and frequency of clinic visits, and 5) self-reported substance use, mental health, and quality of life measures at 1, 2, 4, 8, and 12 weeks. We hypothesize that patrons assigned to the library-facilitated telehealth group will take up buprenorphine, pick up their buprenorphine prescriptions from the pharmacies, take buprenorphine after two weeks, and adhere to medical appointments more than those in the control (in-person clinic visits only) group.

In the study’s formative stages of intervention development, study staff conducted interviews and focus groups with unstably housed library patrons and staff (i.e., security, librarians, outreach workers, and parents) across four libraries in San Diego to assess the feasibility and acceptability of the planned intervention. The findings from the interviews and focus groups informed the design of the telehealth intervention at the libraries.

### Pilot study design

This multisite study employed a parallel-arm randomized controlled trial design with participants allocated to library-facilitated telehealth visits for buprenorphine treatment (BBB intervention) or standard-of-care clinic-based or telephone visits (control). The participants were interviewed before randomization (baseline) and again at 1, 2, 4, 8, and 12 weeks (follow-up) to assess participant characteristics and study outcomes. The study staff also conducted weekly urine drug screenings in the libraries and periodically reviewed medical records and pharmacy pick-up data as part of the study design. An Institutional Review Board at San Diego State University approved this study.

### Study setting

As part of the randomization plan, the pilot study began with the San Diego Central Library, a nine-floor public library in downtown San Diego (a high school occupies two floors). The library has multiple conference rooms and study rooms that the public can reserve.

Halfway through the targeted recruitment number of 20 per library, we began using a second public library site, the Mission Valley Branch Library, to recruit and provide telehealth for this study. The Mission Valley branch allowed us to test the feasibility and acceptability of library-facilitated telehealth in multiple indoor/outdoor library settings. The branch is smaller and less central than the downtown Central library location. Nevertheless, it is next to public transportation (trolley, a metropolitan commuter train) and the San Diego riverbed, where many persons experiencing homelessness have camped. Like the downtown Central library, drug overdoses have been observed at the Mission Valley branch.

Before this study, Father Joe’s Villages - Village Health Center, a federally qualified healthcare center, had already implemented a protocol for free, same-day medication for opioid use treatment. FJV also had prior experience offering telebuprenorphine modalities since the COVID-19 pandemic. However, FJV’s telebuprenorphine program is not well known, has limited access, and has never been tested with public libraries until now. The health center is connected to St. Vincent de Paul Village (Father Joe’s Villages) with comprehensive housing and food services in a downtown San Diego location where individuals experiencing homelessness congregate. It is located 0.7 miles from the downtown Central Library and 7.3 miles from the Mission Valley Library.

### Eligibility criteria

Individuals were eligible for this study if they met the following criteria: 1) reside in San Diego County, California; 2) currently unstably housed (living in a shelter, on the streets, in a car, staying with friends or family, in temporarily rented spaces (e.g., motel), abandoned building, in a tent or otherwise unstably housed, e.g., evicted during the past month); 3) self-reported deliberate use of opioids (confirmed by urinary drug screening onsite at the library) within the last 30 days/three months or as determined eligible for buprenorphine by the medical provider at the clinic (i.e., individuals were not eligible if they only reported unintentional use of opioids, for example, when other drugs used were found to be adulterated with fentanyl); and 4) not currently receiving medications for opioid use disorder (such as methadone, buprenorphine, naltrexone) from another medical provider; and 5) had the desire to quit or reduce opioid use by taking buprenorphine.

### Screening and enrollment

Participants were recruited via flyers posted at reference desks and kiosks/bulletin boards at the San Diego Central and Mission Valley libraries. Nonlibrary personnel were not allowed to approach patrons inside the library to respect their privacy. Therefore, in-person encounters within the libraries occurred only when a patron approached the research staff. The research staff sat in reserved library conference rooms. At the Mission Valley library, tables were set up outside and inside the entrance to advertise the study. At he downtown Central Library, a librarian occasionally introduced the research study to the public announcement system to refer patrons. Social work student interns formally working at the library also referred to patrons who came to see them for other needs. In addition, patrons approached the library front desk or security to inquire about the study after seeing a flier or overhearing about the study from others.

Trained research staff also prescreened interested individuals for enrollment in the study during outreach outside the library. Recruitment around the perimeter of the libraries was sometimes necessary to spread the word about the research study. Furthermore, because the Mission Valley library’s location is not central to a commercial district, study staff recruited participants in the immediate areas of the two metropolitan transit system trolley stops closest to the Mission Valley library and along trails above the San Diego riverbed where many unhoused individuals camped.

Unstably housed individuals who expressed an interest in obtaining buprenorphine to treat their current opioid use were invited to a reserved public library room for screening. In-person screenings were then conducted by research staff via a computer-assisted questionnaire to assess eligibility. The researchers obtained written informed consent from the participants to access their FJV Health Center medical record data about their medical appointment adherence for opioid use disorder and drug screenings onsite. The participants also consented to researchers searching a federal CURES database to confirm whether the participants picked up their controlled medications from the pharmacy. The participants indicated their preferred language for communication (English or Spanish). Researchers who were fluent in the selected language then reviewed the corresponding consent forms, also in their preferred language, with the participants. Enrollment began in June 2023.

For both the library-facilitated telehealth and control arms of the study, patrons were not fully enrolled in the RCT until they attended their first medical buprenorphine appointment in person at the FJV Health Center and were confirmed by the medical provider to be eligible for buprenorphine.

Federal legislation expanded physicians’ ability to initiate and maintain pharmacological treatment of opioid use disorder via telehealth (i.e., without an in-person medical evaluation) during the COVID-19 emergency “stay-at-home” public health policies. With increasing evidence supporting the use of telehealth and its benefits in reducing barriers to buprenorphine access, an extension to use telehealth instead of in-person clinic visitation was granted. Then, it was codified into a permanent law [56]. However, this changing regulation was implemented as this pilot study was underway.

### Randomization and intake

After eligibility screening from an FJV Health Center buprenorphine provider, the study staff enrolled and randomized participants into one of two study arms. Both library telehealth and control group participants then completed interviewer-administered surveys and follow-up interviews over 12 weeks at the libraries. The study staff contacted all patrons in both study arms, including those who missed appointments or did not return to participate in the research study at the libraries, to help reconnect them to the research study.

### Study materials and incentives

For each baseline visit and follow-up visit for library-facilitated telehealth and control arms, all participants completing assessments with the library researcher staff received $20 cash (gift card optional), transportation day passes per library visit with the researcher, and snacks/drinks.

### Intervention conditions

For library-facilitated telehealth sessions, the participants attended buprenorphine treatment follow-up appointments in the libraries’ private study rooms. In some situations, participants elected to be interviewed outside. Efforts were made to keep appointments private in locations not within the hearing distance of others. Both libraries had a tablet on a telehealth floor stand in a private study room for this study.

The libraries made a computer tablet available on a heavy platform (to prevent theft) that could be placed in a library study room. The tablet was another alternative for patrons needing an ID card to check out library laptops.

FJV medical providers used a national care standard (either in-person visits or over telehealth) to provide medication adherence counseling and education (e.g., how to take buprenorphine) and provided information about what to expect, appointments, and treatment goals (e.g., relapse prevention, health/mental health referrals).

Researchers assisted with making follow-up appointments with the FJV Health Center for control and telehealth library groups, and research or library staff assisted patrons with logging on to their telehealth appointments.

As part of usual care for participants in both study arms, participants were expected to attend the FJV Health Center in person when asked to perform a follow-up urinary drug test at the clinic. The participants in the library-facilitated telehealth arm were not forced to use the telehealth option solely; they could still attend in-person medical visits (provide clinic urine samples), AOD counselor visits, or support groups at the clinic. However, as described below, the control group was offered library-facilitated telehealth only once they completed their 12-week study.

### Control condition

Participants in the control condition received the same medical treatment in phases on an individual case basis, case management referrals by research staff, research incentives, and face-to-face follow-ups by the researchers at the library as the library-facilitated telehealth condition. The only difference between the study arms was that control participants were not offered library-facilitated video sessions during the 12-week participation in the study. However, after completing the 12 weeks of the study, the control participants were provided with the option of library-facilitated telehealth.

### Data collection

#### Baseline and longitudinal assessments

Following an FJV Health Center physician’s buprenorphine prescription, research staff tracked the participants’ buprenorphine medication and medical appointment adherence through urinary drug screenings and interviews conducted at public libraries. All participants were invited to return to the library weekly for urinary drug screenings and to complete follow-up survey assessments at weeks 1, 2, 4, 8, and 12. Baseline and follow-up assessments were completed in reserved library study rooms.

The 60-minute baseline survey captured the following domains: sociodemographics, behavior, social support, telehealth utilization and comfort, library usage, substance use behaviors, overdose experiences, and treatment history (see Table 1). The content of the follow-up surveys covered the exact domains, with the exception of static domains such as history and demographics. The follow-up survey was shorter (30 minutes) and included questions surrounding buprenorphine uptake, healthcare utilization, and barriers and facilitators to use. The follow-up questions also asked the participants to recall their experiences since their last visit with the research team in the library.

The urinary strips used to assess the effects of buprenorphine, fentanyl, and other drugs were dipsticks. The participants used library bathroom facilities to collect their weekly self-administered urinary drug screening tests at the libraries. Patrons took the urinary drug test strips (for buprenorphine, fentanyl, and other drugs) and a plastic cup to the library bathrooms and returned the test strips in a clear hazard bag for the study staff to record in a deidentified log. After each visit with research staff, participants received 20 dollars cash and a Metro Transit System pass(es) as reimbursement for their time and travel needs to and from their clinic or library appointments.

We anticipate that many participants would not have a cellular phone or reliable phone access, as phones were often lost, stolen, or run out of minutes due to the individual’s economic vulnerabilities, which exposed them to theft from other individuals, a lack of income to pay the bill, and “sweeps” or forcible displacement from law enforcement. For follow-up, the research staff obtained alternate phone numbers and emails of other individuals who could locate the participants. However, for the most part, participants knew the days and times the research staff would be at the library and would simply show up at those designated times (two or three days per week). Researchers also kept library staff and security staff informed of the study location within the library and times of researcher availability to facilitate follow-up meetings with participants.

#### Outcome ascertainment and postintervention assessments

Intervention outcomes determined the feasibility, acceptability, and retention of the library telehealth intervention at baseline and at 1, 2, 4, 8, and 12 weeks on 1) weekly urine drug screenings (for buprenorphine uptake and other drugs); 2) buprenorphine prescription pick-ups; 3) clinic visits; and 4) self-reported measures (e.g., substance use, mental health, and quality of life). (see Table 2).

At the end of the participants’ 12-week participation in the study, research staff interviewed participants about their experience with library telehealth and the acceptability of BBB study participation. To avoid potentially biased responses, interviews were conducted with study staff not involved in the person’s care. All participants in the library-facilitated telehealth and control groups completed a posttreatment satisfaction survey regardless of whether they had sustained treatment. Data are used to assess participant satisfaction and identify areas for improvement. The investigators also interviewed the medical providers and library staff involved in the study to obtain feedback about their experiences with the study protocol.

#### Data tracking and management

For tracking, the researcher assigned each participant a unique identifier number, and the study staff recorded and maintained each session’s date, time, and location. The researchers used an electronic tracking sheet to record drug screening results (buprenorphine, fentanyl, and other substances) collected onsite from the libraries for the research study. They also recorded participants’ receipt of incentives, study staff initials, and the type of assessment they administered (baseline or follow-up). Notes with deidentified data were also recorded in the spreadsheet.

Additionally, participant medical appointment attendance, clinic urinary drug screening outcomes, and CURES database-derived pharmacy pickup data were recorded on a separate HIPAA-compliant database shared only with the FJV Health Center in the study and selected research staff.

#### Sample size and planned data analyses

Our target sample size for the trial was 40 participants, although we allowed for a maximum of 60 participants to account for attrition. The sample size provides adequate precision for effect size estimates, although it is not statistically powered to assess the efficacy of primary outcomes.

Buprenorphine uptake (the proportion who picked up and took buprenorphine at initiation confirmed by the CURES database pharmacy pickup records and urinary drug screenings) and medical appointment adherence (retained in treatment for two or more weeks confirmed by health records) will be divided by the number prescribed and scheduled, respectively. Adherence estimates from this study will also provide the basis for power calculations in the design of future efficacy trials. Quantitative data analysis consists of univariate statistics to describe the entire study sample and by study arm, the prevalence of individual responses, and the prevalence of treatment uptake and adherence. We will conduct an intent-to-treat (i.e., per-randomization) analysis following CONSORT guidelines to explore potential differences in the outcomes between the study arms. Specifically, we will use regression models testing each outcome separately (using the appropriate test depending on the outcome distribution, e.g., logistic regression with binary outcomes). These models treat the intervention condition (library-facilitated telehealth intervention vs. treatment-as-usual control) as the exposure in unadjusted models, and we include potential covariates (e.g., library location, age, sex, race/ethnicity) in adjusted models.

#### Timeline and dissemination plans

**Figure 1:**
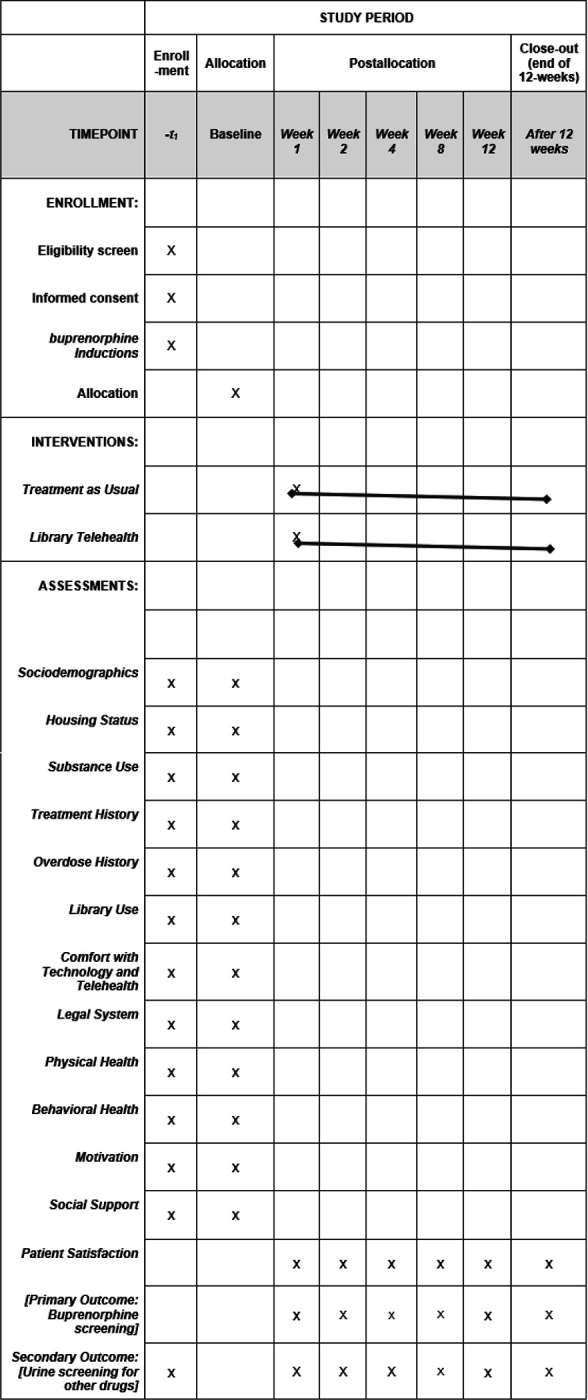
Schedule of enrollment, interventions, and assessments*

The results from the BBB study will be disseminated widely to organizational, clinical, and library partners; community networks; and, nationally, substance use and library information science conferences in consultation with our community partners. Summary findings for community members (nonscientists) will be written following best practices for providing medical information to the public [64]. We will share results with our research partners and stakeholders (e.g., County) via open-access peer-reviewed publications, reports, news media outlets, and conference and community presentations. We aimed to eliminate barriers to future phase III efficacy trials by including residents, patients, providers, and library inputs before the study pilot. We intend to disseminate this information to these groups through these outlets. Effect size estimates may also inform subsequent intervention research, including a fully powered efficacy trial, with this population and setting (see Fig. 1).

## Discussion

Our team of researchers and service providers has learned several vital lessons from the design and implementation of this community-based RCT to improve buprenorphine uptake among unstably housed persons with opioid disorders in San Diego County. This study addresses barriers to buprenorphine uptake by working through the San Diego Public Libraries and FJV Health Center’s federally qualified healthcare center to develop library telehealth interventions for unstably housed library patrons. In partnership with the researchers, two libraries adapted the FJV Health Center’s free telebuprenorphine treatment to their space with available resources. By meeting unhoused people in locations where they already are and providing care regardless of medical coverage, this protocol increases access to buprenorphine to unhoused people, moving beyond overdose prevention measures only to provide follow-up treatment access via public libraries.

On the basis of our prior work, we anticipate that confidentiality issues are important to maintain within the library. At first, we did not inform the library security staff about the project because of the library’s effort to protect participant identities. However, at one point early in the study, security confronted a participant in the bathroom. At the same time, the participants met us in the library, which inevitably led to security in finding out about the study. In retrospect, informing security was necessary to navigate situations where patrons had warnings or were previously suspended from the library.

The refinement of our study protocol includes responsiveness to field experiences and lessons learned as the study was rolled out. Initial challenges included participants arriving for screening and baseline assessments in the library but not attending their initial in-person health visit at the clinic to be fully screened and enrolled. To overcome this, we told participants to enroll in the study by seeing the medical provider first before returning to see the researchers weekly in the library. The screening out of participants who only used stimulants further helped determine eligibility for the study.

Another challenge was selecting an appropriate second library to test a different setting (apart from the large downtown urban library). Of the four libraries we partnered with before conducting the RCT, we considered one in a mainly Latinx neighborhood of San Diego. However, we decided to cultivate the relationship with the community for future consideration instead because 1) the community surrounding the library was improving the area, and some of the focus group participants wondered if the project would attract unwanted patrons who were not yet using the library in high numbers and 2) key informant community members said we would need to proceed carefully with any participants not from the community coming to their library for the study because they could become targets of territorial gangs. Therefore, we selected the Mission Valley branch library, which previously had many patrons overdosing on their premises. The Mission Valley branch has experienced overdoses because of its location above the San Diego riverbed, where many unhoused individuals stay, and its proximity to the public transportation trolley line.

Finally, we anticipated that participants could access telehealth at the library independently. However, it became clear that a social worker or researcher could best facilitate library-facilitated telehealth for buprenorphine for most of the unstably housed participants in our study who had opioid use disorders. Many living on the street without a phone preferred drop-in library-facilitated telehealth appointments, which the research staff facilitated with the clinic partner through a library phone and computer tablet on the same day the participant saw the research staff there. The librarians also helped allow participants to enter the library study room, where the tablet on a stand was kept. In the case of the smaller library, a phone is also available for the medical provider to call to indicate that they are ready for the video visit.

Although barriers remain, such as individuals needing phones for us to follow up, we set consistent days and times at the library so that participants could drop in without a set appointment. We held the same hours on the same days of the week with signage indicating our presence. This enabled participants to quickly follow up at the study site if they were without communication devices. We also learned that although many participants may not have had a phone consistently, some typically had an email address they checked regularly and could receive reminders via this method.

Other barriers the population encounters include needing a photo identification (ID) to pick up their medication at the pharmacy or checking out a library laptop. Several participants without an ID said that their belongings had been discarded during police sweeps or were stolen or lost. Nevertheless, we helped them overcome these with an agreement with the libraries to allow them to use the tablet on a telehealth floor stand without a library card or one that requires a photo ID. We referred patrons to places that can help them obtain an ID and informed them that they can ask a trustworthy friend or family member with an ID to help them pick up their prescriptions, although many living on the street lack such support.

Overall, our design and changes in response to issues as they arise have contributed to the success of the BBB pilot study’s implementation. For example, we provided bike locks and snacks so that unstably housed participants could comfortably see us in the library without worrying about their belongings or hunger. We often provided pizza for the participants, as they completed twelve weeks of the study and gave them a certificate of completion. Although participants sometimes relied on the twenty-dollar weekly incentive for their time doing follow-up assessments, the moderate amount did not appear coercive. Nevertheless, it may motivate their initial interest and retention in the study. Through consistent communication, we fostered a sense of community and increased morale among patrons and staff. The study design enables library-facilitated telehealth to be sustained with minimal extra work for libraries and clinics.

## Conclusion

In conclusion, our team found that implementing a community-based pilot study to increase buprenorphine uptake among unstably housed people using opioids is feasible. The pilot study highlights the importance of building collaborative relationships on the basis of mutual respect and trust to address unexpected challenges. Public health and social work initiatives aimed at socially and structurally marginalized populations, such as unhoused library patrons, can benefit from partnering with community-based organizations such as public libraries.

By testing library telehealth for buprenorphine uptake and retention, this study demonstrates how unstably housed persons may receive buprenorphine care innovatively with minimal clinical/nonclinical requirements (e.g., low-barrier induction) in community-based settings. Ultimately, we hope that findings and lessons from this pilot study will support the adoption of library-facilitated telehealth interventions through public libraries or other community-based settings that serve unstably housed people impacted by substance use and addiction.

## Figures and Tables

**Table 1:** Survey covariate measures

Topic/Variable	Covariate Measure	Link to survey instrument
**Outcomes**		
Buprenorphine uptake/adherence	“How often do you take it? What is the dosage? Are you taking it correctly (as prescribed)? If not, why?” “Did you run out of your prescription before getting a refill?” “Have you stopped taking Suboxone?”	study generated (augmented by biomarkers/: urinary drug screens at the library and clinic, pharmacy pickup data from the federal CURES database, and clinic medical record data for appointment adherence)
Substance Use	NIDA-Modified ASSIST V2.0 [57]	http://www.sbirtoregon.org/wp-content/uploads/Modified-ASSIST-English-pdf.pdf
Alcohol Use	AUDIT-C [58]	https://cde.drugabuse.gov/instrument/f229c68a-67ce-9a58-e040-bb89ad432be4 or https://nida.nih.gov/sites/default/files/pdf/nmassist.pdf
Motivation (e.g., to stop using opioids, to take buprenoprhine)	Readiness Ruler [59]	https://case.edu/socialwork/centerforebp/resources/readiness-ruler
Depression and Anxiety	PHQ-4 [60]	https://www.oregonpainguidance.org/app/content/uploads/2016/05/PHQ-4.pdf
**Background**		
Childhood Trauma Experiences	ACES [61]	https://www.acesaware.org/wp-content/uploads/2022/07/ACE-Questionnaire-for-Adults-Identified-English-rev7.26.22.pdf
Social Support	MOS Social Support Survey [62]	https://pubmed.ncbi.nlm.nih.gov/2035047/
Experience with Technology	Adapted from Dr. Garfein’s VDOT study [63]	Garfein, et al. (2018). Tuberculosis Treatment Monitoring by Video Directly Observed Therapy in 5 Health Districts, California, USA. *Emerging infectious diseases, 24*(10), 1806–1815. https://doi.org/10.3201/eid2410.180459
Library Usage	Questions around individuals use of the library (e.g., time spent visiting, activities at library, frequency of visits)	study generated
OUD Treatment Histories	Questions eliciting information on prior pharmacological or psychological treatment of opioid use disorder (e.g., methadone, counseling)	study generated

**Table 2:** BBB intervention targets, strategies, and specific activities

Intervention targets	Intervention strategies	Examples of specific activities	Format
Awareness of buprenorphine availability	Basic buprenorphine education, increase in knowledge of service providers	General information on buprenorphine availability at Father Joe’s Villages and in the San Diego region	Personalized discussion, resource list
Apprehension toward buprenorphine*	Basic buprenorphine education, increase knowledge of health	· General background on buprenorphine treatment (e.g., targeting of opioids) · Facts about buprenorphine · Buprenorphine treatment information	Brief educational information sheets
Limited MOUD treatment skills	Problem-solving	· Identifying challenges relevant to individuals in continuing through treatment (e.g., personal identification for pharmacy, contact information for reminder)	Personalized discussion to address barriers
Limited telehealth behavioral skills	Modeling	· Modeling process of logging in to the telehealth system · Education on what medical professionals to expect and when (e.g., nurse followed by the doctor)	Demonstration

* **Buprenorphine Apprehension**. Though individuals wanted to try buprenorphine to help them treat their opioid use, many expressed fear of possible significant side effects, such as precipitated withdrawal. Others had the perception that MOUDs would not work for them, and others still did not know what buprenorphine was.

## Data Availability

Not applicable
